# Epicuticular wax of sweet sorghum influenced the microbial community and fermentation quality of silage

**DOI:** 10.3389/fmicb.2022.960857

**Published:** 2022-07-29

**Authors:** Wei Tang, Longxing Liao, Yu Xiao, Jianrong Zhai, Hang Su, Yingjie Chen, Yanjun Guo

**Affiliations:** ^1^College of Grassland Science, Qingdao Agricultural University, Qingdao, China; ^2^College of Agronomy and Biotechnology, Southwest University, Chongqing, China; ^3^College of Animal Science and Technology, Southwest University, Chongqing, China

**Keywords:** bloom, fermentation quality, microbial community, silage, sweet sorghum

## Abstract

Epicuticular wax, as secondary metabolites (hydrophobic compounds) covering plant surface, plays important roles in protecting plants from abiotic and biotic stresses. However, whether these compounds will influence fermentation process of silage is still not clear. In this study, two sweet sorghum cultivars with varying epicuticular wax on sheath (bloom), Yajin 2 (YJ, less bloom), and Jintian (JT, dense bloom), were harvested at flowering and maturing stages, and ensiled with or without bloom, aiming to evaluate the effects of bloom on fermentation quality, feed nutrition and microbial community. The bloom was collected manually with de-waxed cotton and extracted with chloroform. The results showed that the bloom reduced the concentrations of water-soluble carbohydrate and crude protein of the two cultivars at both stages, reduced lactic acid (LA) for YJ at both stages and for JT at flowering stage, and increased LA for JT at mature stage. The α-diversity of bacterial communities of the silage fermentation with bloom was significantly lower than that without bloom. Bloom increased the abundance of *Lactobacillus*, reduced that of *Bacillus* and *Weissella*, and significant correlations were observed between fermentative qualities and bacterial abundances. However, decreased diversity of bacterial community and the contents of LA implied that shifts in bacterial community might exert negative effects on silage fermentation. Our results suggest that bloom wax could alter the microbial community composition of ensiled sweet sorghums, which thus influence the fermentation qualities.

## Introduction

Cuticular waxes are mixtures of hydrophobic compounds covering the surface of land plants (Jeffree, 2006). These compounds play important roles in protecting plants from abiotic and biotic stresses. However, for forage crops such as corn and sorghum, the existence of cuticular wax may influence forage qualities, and thus its feeding value. An early study by Cummins and Dobson (1972) found that the sheath bloom reduced *in vitro* dry matter (DM) digestibility of sorghum. Silage, which is fermented mainly by lactic acid bacteria (LAB) existing on plant surface, is one of the important methods widely used in forage production. However, whether the cuticular wax of forage crop will influence its fermentation quality is still not clear.

Sweet sorghum (*Sorghum bicolor* Moench) is an important annual forage crop widely distributed all over the world (Bai et al., 2021; Ran et al., 2021; Wang et al., 2021). As a C_4_ plant, its high yield, sugar-rich stalks and high resistance to adverse environments make it the best choice for forage production in arid and semi-arid areas where water shortage limits silage corn production (Huang et al., 2020). Meanwhile, sorghum is also one of the crops with higher wax coverage. Due to high wax accumulation, the sheath of some sorghum cultivars present heavy bloom (whitish) on their surface (Peterson et al., 1982). The amounts of cuticular wax of sorghum can reach 9–15 μg/cm^2^ on leaf and 113 μg/cm^2^ on sheath (Jordan et al., 1984; Jenks et al., 2000). Detailed chemical analysis of sorghum wax showed that sheath wax was dominated with acids (42.8%), whereas leaf was dominated with alkanes (28.4%) and aldehydes (28.4%), and stem with aldehydes (80.8%) (Xiao et al., 2020).

Previous studies have shown that plant cuticular waxes can be applied in food processing (de Freitas et al., 2019), post-harvest storage (Mattos et al., 2017), pharmacological activities (Szakiel et al., 2012), and disease control (Favaro et al., 2020). A study using sorghum wax also indicated that sorghum sheath bloom would reduce the growth of *Penicillium* but unchange *Alternaria alternata* (Xiao et al., 2020). By scraping the sheath bloom from sweet sorghum, Cai et al. (2013) found that the addition of wax powder inhibited the acetone-butanol-ethanol fermentation using *Clostridium acetobutylicum* ABE1201. The very-long-chain aldehydes on maize leaf benefited the fungal prepenetration of *Blumeria graminis* f.sp. *hordei*) (Hansjakob et al., 2010). These results suggested that the compositions of sorghum blooms might differ in their efficiency of biological activities and thus the silage microbes. However, no direct evidences prove how bloom will influence silage microbe.

The LAB strains differ greatly among plant species and cultivars (Puntillo et al., 2020), and thus their fermentation efficiency (Nazar et al., 2020). For example, LAB strains isolated from corn, forage paddy rice, sorghum and alfalfa silages differed in their abilities to produce lactic acid and fermentation quality (Pang et al., 2011). Dong et al. (2020) also reported that the sweet sorghum bagasse silage inoculated with isolated lactic acid bacteria achieved low pH value, high levels of lactic acid and water-soluble carbohydrates (WSC) content. Ferulic acid esterase-producing LAB might partially ameliorate the negative effects of high temperature and improve the fermentation quality of sorghum silage (Xie et al., 2021). Studies have also shown that the plant secondary metabolites will influence the ensiling characteristics (Popp et al., 2016). For example, intrinsic tannins affected ensiling characteristics and proteolysis of *Neolamarckia cadamba* leaf silage by largely altering bacterial community (He et al., 2020). Therefore, the cuticular wax on sorghum might influence the growth of LAB and subsequently the fermentation quality.

To clarify how the sheath bloom influences the silage quality, in the current study, we scraped the bloom of sweet sorghums in their early flowering and maturing stages, analyzed the effects of bloom on silage fermentation quality and microbial community, and determined the relationship between ensiled sorghum properties and microbial diversity and compositions. This is the first report elucidating how the bloom will influence silage quality and microbe communities for forage crops. The main objective of the current study is to provide suggestions for sweet sorghum breeding, when breeding sweet sorghums with high wax coverage for drought resistance, the influence of cuticular wax on its forage quality is also needed to be evaluated.

## Materials and methods

### Ensiling of sweet sorghum

The sweet sorghum (*Sorghum bicolor*) cultivars selected in this study were Yajin 2 (YJ) and Jintian (JT). The two cultivars differed in bloom content (YJ was bloom-depleted type and JT was bloom- enriched type). The seeds were surface sterilized in 10% H_2_O_2_ for 10 min, washed under tap water, and then were planted in Experiment Farm of Southwest University, located in Beibei district, Chongqing, China.

The growth stages of the two cultivars were similar (Supplementary Table 1). At stages of early flowering (30% of plant flowering, BBCH scale 63, general) and mature (seed dough stage, BBCH scale 85, general), the bloom on sheath of plants were manually collected from sorghum stalks using de-waxed cotton. Then the scraped powders were extracted with chloroform, filtered with glass wool, dried under nitrogen gas, and stored at 4°C with oxygen free in the tank for cuticular wax analysis (Table 1). Later, the sweet sorghum plants (sheath with bloom (bloom+) and without bloom (bloom−)) were harvested with a stubble of 1 cm. The amounts of bloom removed from sheath differed between cultivars. The sweet sorghums were chopped into 2 cm pieces, which were packed into polyethylene plastic bag (250 mm × 350 mm) and vacuum-sealed, and then were incubated at 25–30°C for 45 days until samples were used for silage quality analysis. Each bag was filled with 150 g sorghum and each treatment replicated three times. At silo opening, the top and lower layers of silages in the silo were discarded, and the remaining silages were homogeneously mixed for the analysis of chemical composition, fermentation profile, and microbial community.

**TABLE 1:** Cuticular wax profiles on organs of sweet sorghum and the amount of bloom collected from sheath.

Organ	Wax composition	Flowering stage (μg cm^–2^)		Maturing stage (μg cm^–2^)	
			
		Yajin	Jintian	Yajin	Jintian
Leaf	Acids	0.65 ± 0.02	0.97 ± 0.12	0.18 ± 0.02	0.37 ± 0.01
	Aldehydes	–	–	–	–
	Alkanes	1.51 ± 0.34	1.39 ± 0.17	0.24 ± 0.00	0.27 ± 0.00
	1°Alcohol	0.51 ± 0.20	0.65 ± 0.14	0.15 ± 0.01	0.26 ± 0.01
	Others	0.54 ± 0.13	0.89 ± 0.19	0.24 ± 0.02	0.29 ± 0.01
Stem	Acids	0.15 ± 0.03	0.49 ± 0.15	0.56 ± 0.03	0.22 ± 0.02
	Aldehydes	4.13 ± 0.66	6.80 ± 0.54	9.42 ± 1.00	7.02 ± 0.73
	Alkanes	0.36 ± 0.08	1.12 ± 0.11	1.05 ± 0.02	0.55 ± 0.02
	1°Alcohol	0.28 ± 0.08	0.93 ± 0.24	0.33 ± 0.01	0.68 ± 0.14
	Others	0.17 ± 0.02	0.48 ± 0.15	0.53 ± 0.11	0.74 ± 0.03
Sheath	Acids	7.07 ± 0.23	10.19 ± 1.42	1.50 ± 0.06	0.93 ± 0.04
	Aldehydes	–	–	–	–
	Alkanes	0.45 ± 0.09	0.40 ± 0.03	0.05 ± 0.00	0.05 ± 0.00
	1°Alcohol	0.77 ± 0.10	0.42 ± 0.05	0.05 ± 0.01	0.05 ± 0.00
	Others	0.53 ± 0.07	0.42 ± 0.00	0.17 ± 0.05	0.13 ± 0.02
Amount of collected bloom (mg per 150 g sorghum)^a^		2.85	3.00	1.95	3.15

Others included triterpenoids, sterols, and small amounts of unidentified compounds.

–, undetectable.

^a^The amount of bloom collected from sorghum sheath calculated by the weight of silage.

### Characteristics of ensiled sorghum

The pre-ensiled sorghums and the silage samples were dried at 65°C for 48 h, weighed, and then grounded with a sieve (1.0 mm) to determine the contents of crude protein (CP), ether extract (EE), crude fiber (CF), water soluble carbohydrate (WSC), and crude ash (Ash), according to the methods described by Yang et al. (2019). CP was determined by Kjeldahl method, EE was determined by Soxhlet extraction, CF was determined by acid and alkali hydrolysis, WSC was determined by anthrone concentrated sulfuric acid method, and Ash was determined by high temperature burning method.

The fresh ensiled samples (20 g) were extracted with 180 mL distilled water at 4°C for 24 h, filtered through double-layer gauze and filter paper, and then the filtrates were stored in refrigerator at −20°C. The filtrate was used to determine pH, lactic acid, ammonia nitrogen and volatile fatty acids. pH was measured using a glass electrode pH meter. Lactic acids were analyzed using capillary gas chromatography (GC; DB-ffap quartz capillary column, Agilent; Fuli, China). The detailed GC parameters were as follows: oven temperature 110°C; injection port temperature 250°C; detector temperature was 290°C; N_2_ flow rate 32.4 mL min^–1^; H_2_ flow rate 40.0 mL min^–1^; air flow rate 400.0 mL min^–1^; tail blowing 30 mL min^–1^; Split ratio 20:1. The ammonia nitrogen (NH_3_-N) was determined using the method described by Broderick and Kang (1980). The total nitrogen (TN) was determined by Kjeldahl method. Volatile organic acids were analyzed using capillary GC (DB-ffap quartz capillary column, Agilent; Fuli, China) with some modifications to the method described by Darwin et al. (2018). The GC programs were as follows: initial oven temperature 60°C, raised to 100°C at the rate of 4°C/min and kept for 5 min; injection port temperature 200°C; detector temperature was 250°C; nitrogen flow rate 32.4 mL min^–1^; hydrogen flow rate 40.0 mL min^–1^; air flow rate 400.0 mL min^–1^; tail blowing 30 mL min^–1^; Split ratio 30:1.

### Microbial diversity analysis of ensiled sorghum

The total genomic DNA was extracted from silage, and the high variation region of the bacterial 16S rRNA gene v3–v4 was obtained by 338F (5′-ACTCCTACGGGAGGCAGCA-3′) and 806R (5′-GGACTACHVGGGTWTCTAAT-3′). The PCR program was as follows: 95°C for 3 min, 27 cycles (30 s at 95°C, 30 s at 55°C, 45 s at 72°C) with a final extension of 72°C for 10 min and 10°C until being halted. The PCR products were detected by 2% agarose gel electrophoresis, and the correct PCR products were identified and sequenced using Illumina PE300 sequencing platform (Shanghai Meiji Biomedical Technology Co., Ltd.). The original sequences were processed by Meiji Bio Cloud Platform. The RNA-Seq reads were deposited and available at the database Sequence Read Archive at NCBI^1^ under the master accession number of Bioproject PRJNA853257.

### Wax extraction and analysis

The leaves and sheaths (third leaf from the top) and stems (between internode three and four) were sampled from healthy plants growing in fields at flowering and maturing stages, with three replicates for each cultivar. Wax extraction was based on the methods described by Xiao et al. (2020). Surface areas of leaf and sheath were determined by pixel counting the sample photos using the ImageJ software. The stem was regarded as cylinder and its surface areas were calculated using the diameter and height [Surface area (cm^2^) = 3.1415 × diameter × height]. Then, the samples were extracted twice for 30 s with CHCl_3_ containing 5 μg of *n*-tetracosane as internal standard. The two extracts from each sample were then combined and filtered through glass wool, dried under N_2_, and then derivatized using pyridine (20 μL, ≥99%, Sigma-Aldrich) and *N*,*O*-bis(trimethylsilyl)trifluoroacetamide (20 μL, BSTFA + TMCS 99:1, Sigma-Aldrich) at 70°C for 45 min. The excess reagents were dried under N_2_, and re-dissolved in 0.5 mL CHCl_3_ for GC analysis.

The GC analysis was carried out with FULI 9790II (Zhejiang Fuli Analytical Instrument Co., Ltd., Wenzhou, China) using DM-5 capillary column (30 m × 0.32 mm × 0.25 μm) (Beijing Dikema Technology Co., Ltd.). N_2_ was served as carrier gas. The temperature of the column membrane and FID detector was set at 300 and 320°C, respectively. The GC oven was held at 80°C for 10 min, heated at 5°C/min to 260°C, remained 10 min, then heated at 2°C/min to 290°C, and further heated at 5°C/min to 320°C, held for 10 min. Compounds were further detected with a GCMS-QP2010 Ultra Mass Spectrometric Detector (Shimadzu Corp., Kyoto, Japan) using HP-5 MS capillary column (30 m × 0.32 mm × 0.25 μm), and He as the carrier gas. Compounds were identified by comparing their mass spectra with published data and authentic standards. Wax quantification was based on the peak area of internal standard and presented as micrograms per unit leaf area.

### Statistical analysis

The presented data in figures and tables were the average of three biological replicates. Two-way ANOVA was applied to evaluate the influence of cultivars and bloom on fermentation qualities using SPSS software (17.0, Chicago, IL, United States). Then, one-way ANOVA was applied to compare the difference in fermentation parameters between with bloom and without bloom treatments. Differences were regarded significance at *P* < 0.05 according to the least significant difference test. The redundancy analysis (RDA) analysis was applied to analyze the relationship between fermentation qualities and microbial community at the phylum and genus level using Canoco 5. The significance of each variable was defined using Mantel tests (permutations = 999) and significance level was tested by Mantel *R*^2^ statistic and *p*-values. Spearman correlation heatmap was used to show the relationship between bacterial classifications and environmental variables (chemical composition and fermentation products).

## Results

### Wax profiles of sweet sorghums

The cuticular waxes on leaves, stems and sheaths of sorghums were analyzed separately. Overall, leaves were mainly predominated by fatty acids (averaged 26% across two cultivars and two stages), alkanes (31%), and primary alcohols (20%) (Table 1). However, stems were predominated with aldehydes (72%) and sheath with fatty acids (86%). The relative abundance of wax compositions was similar among cultivars and between stages, however, the wax coverage varied greatly between cultivars and stages. For example, leaf total wax (sum of acids, aldehydes, primary alcohols, alkanes, and others) ranged from 1.75 to 2.92 μg cm^–2^ at flowering stage and from 0.63 to 1.11 μg cm^–2^ at maturity stage. Stem wax ranged from 5.09 to 9.82 μg cm^–2^ at flowering stage and from 9.21 to 16.80 μg cm^–2^ at maturing stage. Among the organs, sheath had the highest wax coverage at flowering stage, with sheath wax on and Jintian (JT) reaching 11.43 μg cm^–2^, followed by Yajin 2 (YJ) (8.82 μg cm^–2^). However, the wax coverage on sheath at maturing stage was lower than that at flowering stage, and even was lower than those on stem which increased from flowering stage to maturing stage. Using de-waxed cotton, the amounts of bloom collected from sorghum sheath reached 2.85 and 1.95 mg per 150 g plant for YJ, and 3.00 and 3.15 mg per 150 g plant for JT, at flowering and maturing stages, respectively. No significant difference of chemical profiles could be observed between sheath wax and bloom.

### Characteristics of ensiled sorghum

Besides their difference in wax depositions, the three sorghum cultivars also differed in their chemical properties related to feed qualities, particularly the concentrations of water-soluble carbohydrates (WSC) and EE (Table 2). For example, the WSC in YJ was only 137 g kg^–1^, which was significantly lower than those in JT (200 g kg^–1^) at maturing stage, whereas the concentrations of EE in YJ was significantly higher those in JT at both stages. Compared with flowering stage, overall, the DM and WSC increased, whereas CP, CF and crude ash (Ash) reduced, at maturing stage.

**TABLE 2:** The chemical composition (g kg^–1^ DM) of sweet sorghum straw at flowering and maturing stages.

Stage	Cultivar	DM	WSC	CP	EE	CF	ASH
Flowering	Yajin	17.93 ± 0.40	72.08 ± 2.55	78.59 ± 0.48	81.00 ± 1.00	347.67 ± 1.86	79.67 ± 0.44
	Jintian	17.33 ± 0.46	75.87 ± 3.35	75.02 ± 0.76	73.97 ± 1.13	371.54 ± 1.44	79.50 ± 1.76
Maturing	Yajin	23.01 ± 1.58	137.01 ± 4.14	48.47 ± 0.54	80.31 ± 1.20	253.25 ± 1.91	66.50 ± 0.14
	Jintian	26.89 ± 0.91	200.23 ± 4.91	45.84 ± 0.53	75.62 ± 0.70	262.49 ± 1.70	74.42 ± 0.22

DM, dry matter; WSC, water-soluble carbohydrates; CP, crude protein; EE, ether extract; CF, crude fiber.

After the sorghums were ensiled for 45 days, the chemical compositions were evaluated. Overall, the cultivar, sheath bloom and their interactions significantly influenced the chemical compositions of ensiled sorghums (Table 3). For example, bloom significantly reduced the concentrations of WSC for all cultivars at both stages, excepting for an increase for YJ at flowering stage. A decrease of CP in sorghum with bloom was also observed for all cultivars at two stages, excepting for insignificant changes for JT at flowering stage. Bloom reduced the concentrations of EE in JT at flowering stage, whereas increased that in YJ at maturing stage. When compared with the sorghum without bloom, the sorghum with bloom overall negatively influenced the quality of ensiled sweet sorghum, particularly WSC and CP.

**TABLE 3:** The chemical composition (g kg^–1^ DM) of sweet sorghum silage at flowering and maturing stages.

Stage	Cultivar	Bloom	DM (%)	WSC	CP	EE	CF	ASH
Flowering	Yajin	−	17.15 ± 0.17a	2.97 ± 0.18b	53.96 ± 2.92a	104.60 ± 0.96a	364.09 ± 1.16a	82.33 ± 5.04a
		+	16.93 ± 0.47a	5.33 ± 0.31a	44.48 ± 2.63b	73.27 ± 0.31b	375.75 ± 8.65a	85.33 ± 2.91a
	Jintian	−	15.33 ± 0.11a	4.24 ± 0.00a	51.77 ± 1.46a	92.57 ± 5.02a	336.99 ± 4.82a	80.34 ± 2.91a
		+	15.06 ± 0.38a	2.79 ± 0.18b	51.04 ± 0.73a	98.00 ± 3.21a	346.33 ± 4.33a	87.00 ± 2.08a
		**Analysis of variance**						
	Cultivar		33.42***	9.81*	1.05	4.41	27.02***	0.002
	Bloom		0.59	5.01	5.77*	18.35**	3.73	2.00
	Cultivar × Bloom		0.11	88.26***	4.23	36.96***	0.05	0.29
Maturing	Yajin	−	24.13 ± 0.52a	25.25 ± 1.32a	50.58 ± 0.88a	71.18 ± 4.99b	345.96 ± 3.84a	88.37 ± 1.27a
		+	23.66 ± 1.10a	15.55 ± 0.76b	46.62 ± 0.88b	92.14 ± 2.99a	330.71 ± 10.56a	87.57 ± 1.37a
	Jintian	−	24.19 ± 0.42a	39.81 ± 1.27a	51.46 ± 0.00a	72.72 ± 0.46a	338.46 ± 4.38a	72.00 ± 1.53b
		+	23.82 ± 0.17a	34.54 ± 1.52b	44.42 ± 1.59b	70.16 ± 1.05a	353.40 ± 5.61a	82.72 ± 1.22a
		**Analysis of variance**						
	Cultivar		0.027	180.6***	0.43	11.85**	1.30	61.74***
	Bloom		0.43	36.0***	29.8***	9.61*	0.001	13.5**
	Cultivar × Bloom		0.01	3.15	2.33	15.7**	5.15	18.2**

DM, dry matter; WSC, water-soluble carbohydrates; CP, crude protein; EE, ether extract; CF, crude fiber. −, without bloom; +, with bloom. Values followed by different lowercase letters in the same column within each cultivar are significantly different at P < 0⋅05 according to the least significant difference test. *P < 0.05, **P < 0.01, ***P < 0.001.

The fermentation products were also influenced by cultivar, bloom and their interactions (Table 4). The bloom had no significant influence on the concentrations of acetic acid (AA), propionic acid (PA) and butyric acid (BA) at flowering stage. However, at maturing stage, an increase of AA and BA for YJ was observed. The bloom increased the ratio of ammonia nitrogen in total nitrogen (AN/TN) for YJ at flowering stage, reduced that for YJ at maturing stage, and unchanged for the others. And a higher AN/TN was observed at maturing stage than at flowering stage. The bloom significant influenced on the concentrations of lactic acid (LA) at maturing stage. Bloom decreased the LA for both cultivars.

**TABLE 4:** The pH and fermentation products (%) and ammonia-*N*/total *N* of sweet sorghum silage at flowering and maturing stages.

Stage	Cultivar	Bloom	pH	LA	AA	PA	BA	AN/TN
Flowering	Yajin	−	3.98 ± 0.02a	4.34 ± 001a	1.28 ± 0.01a	0.16 ± 0.01a	0.19 ± 0.00a	0.88 ± 0.16b
		+	3.97 ± 0.02a	3.55 ± 0.16b	1.25 ± 0.03a	0.13 ± 0.00a	0.15 ± 0.00b	1.40 ± 0.029a
	Jintian	−	3.79 ± 0.04a	3.27 ± 0.20b	1.32 ± 0.03a	0.10 ± 0.01a	0.11 ± 0.01b	1.19 ± 0.09a
		+	3.75 ± 0.02a	4.66 ± 0.23a	1.39 ± 0.04a	0.14 ± 0.01a	0.16 ± 0.01a	0.98 ± 0.09a
		**Analysis of variance**						
	Cultivar		63.6***	0.01	9.34*	26.3***	22.2**	0.30
	Bloom		0.95	2.86	0.37	0.82	0.89	1.89
	Cultivar × Bloom		0.71	40.1***	2.25	32.8***	37.6***	10.5*
Maturing	Yajin	−	4.16 ± 0.03a	3.60 ± 0.11a	0.61 ± 0.02b	0.13 ± 0.00a	0.31 ± 0.12b	5.38 ± 0.12a
		+	4.06 ± 0.07a	2.57 ± 0.21b	0.97 ± 0.08a	0.14 ± 0.01a	0.51 ± 0.05a	3.33 ± 0.27b
	Jintian	−	3.83 ± 0.02a	7.52 ± 0.74a	0.71 ± 0.05a	0.11 ± 0.00a	0.08 ± 0.01a	8.08 ± 0.33a
		+	3.87 ± 0.03a	4.81 ± 0.13b	0.71 ± 0.01a	0.11 ± 0.00a	0.08 ± 0,00a	8.24 ± 0.40a
		**Analysis of variance**						
	Cultivar		32.7***	61.2***	2.82	8.00*	139.5***	160.7***
	Bloom		0.44	22.5**	16.1**	0.5	12.0**	10.0*
	Cultivar × Bloom		2.37	4.6	15.6**	<0.001	13.7**	13.6**

AA, acetic acid; AN, ammonia nitrogen; BA, butyric acid; LA, lactic acid; PA, propionic acid; TN, total nitrogen. −, without bloom; +, with bloom. Values followed by different lowercase letters in the same column within each cultivar are significantly different at P < 0⋅05 according to the least significant difference test. *P < 0.05, **P < 0.01, ***P < 0.001.

### Bacterial diversities and communities in ensiled sorghum

**FIGURE 1:**
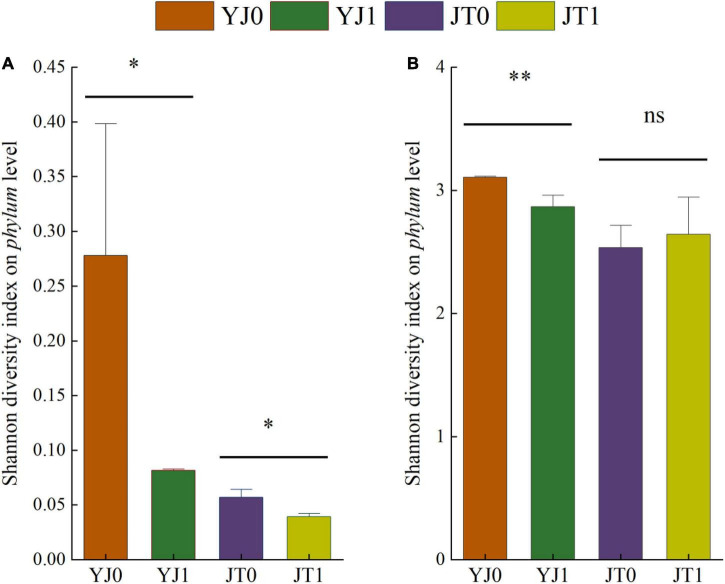
Diversity indices (Shannon) of bacterial community **(A,B)** at phylum level in ensiled sorghum at flowering and maturing stage. All data represented means ± SD. * and ** indicated the level of significance at *p* < 0.05 and *p* < 0.01; ns indicated non-significance. YJ0, Yajin without bloom; YJ1, Yajin with bloom; JT0, Jintian without bloom; JT1, Jintian with bloom.

Differences of bacterial communities in ensiled sorghum were revealed by the comparisons of diversity index (Figure 1). On average, Shannon diversity index on phylum level were higher in silage without bloom than with bloom, excepting for insignificant difference for JT at maturing stage and the bacterial diversity of silage at the maturing stage was higher than that at flowering stage.

**FIGURE 2:**
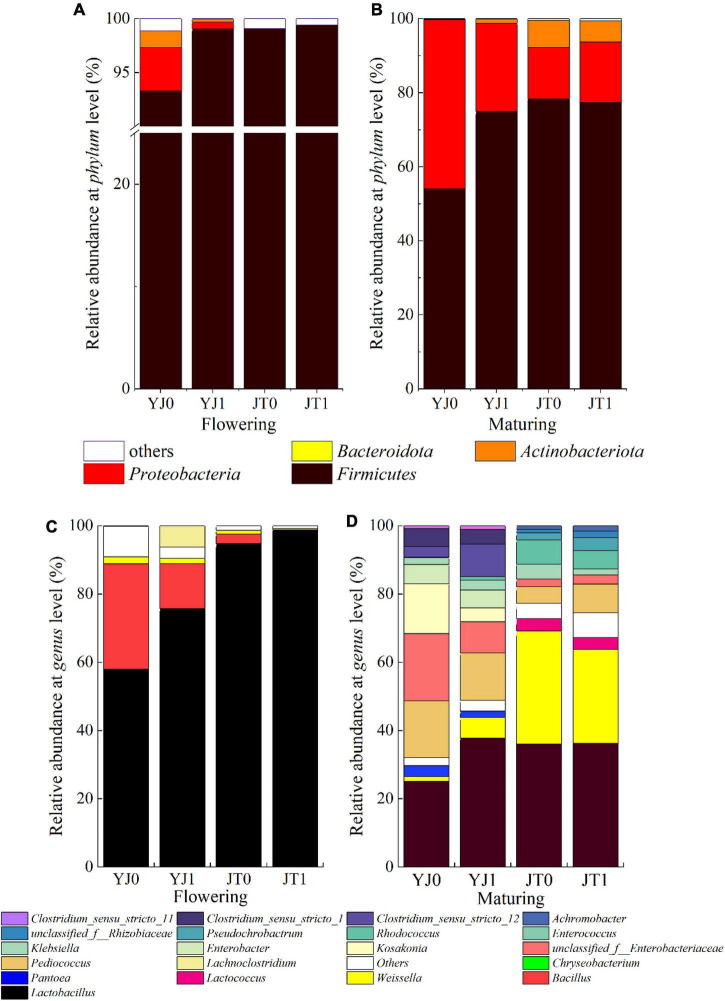
The effects of sheath bloom on the bacterial abundance at phylum **(A,B)** and genus **(C,D)** level in ensiled sweet sorghum at flowing and maturing stages, respectively. YJ0, Yajin without bloom; YJ1, Yajin with bloom; JT0, Jintian without bloom; JT1, Jintian with bloom.

The bacterial community composition was also affected by bloom. Overall, the relative abundance of phylum *Firmicutes* was the highest for sorghum silages irrespective of cultivars and growing stages, accounting for 96.95% (averaged across two cultivars) at flowering stage and 75.77% at maturing stage, followed by *Proteobacterial* accounting for 1.95% at flowering stage and 21.55% at maturing stage (Figure 2A). Bloom relatively increased the abundance of *Firmicutes* but reduced the abundance of *Proteobacteria* for all cultivars at flowering stages (except for JT) (Figure 2A). Compared to flowering stage, bloom reduced the abundance of *Firmicutes* and increased the abundance of *Proteobacteria* at maturing stage (Figure 2B). Bloom also reduced the abundance of *Actinobacteriota* for YJ at flowering stage and JT at maturing stage, and the abundance of *Bacteroidota* at flowering stage.

At the genus level, more obvious difference in microbial abundance was observed between ensiled sorghum with bloom and without bloom (Figures 2C,D). Bloom increased the abundance of *Lactobacillus* (83% averaged across all cultivars), the dominant microbe, for all cultivars at both stages, except for insignificant changes for JT at maturing stage. *Bacillus* was the second largest genus at flowering stage, the abundance (averaged 9.82%) of which was reduced by bloom for all cultivars. No *Bacillus* was detected at maturing stage. *Weissella* was the second largest genus at maturing stage (19.39%), the abundance of which was reduced by bloom from 33.22 to 27.53% for JT. The abundance of *Weissella* at flowering stage was all lower than 4%, which also was reduced by bloom for both cultivars. In addition, compared to flowering stage, there were more bacterial communities detected at the genus levels at maturing stage, such as *Pediococcus*, *Kosakonia*, and *Rhodococcus*.

### Correlations between silage qualities and microbial communities

**FIGURE 3:**
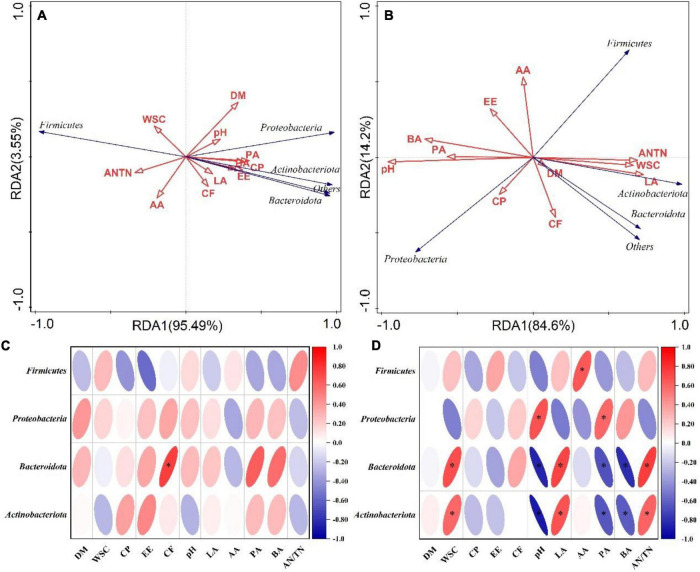
The RDA plots showing the effects of chemical composition and fermentation products on the bacterial community structure at the phylum level at flowering **(A)** and maturing stage **(B)**. Spearman correlation heatmap showed the relationship between bacterial classification at the phylum level at flowering **(C)** and maturing stage **(D)**, and environmental variables (chemical composition and fermentation products). * indicated the level of significance at *p* < 0.05. AA, acetic acid; AN/TN, ammonia-N in total N; BA, butyric acid; CF, crude fiber; CP, crude protein; DM, dry matter; EE, ether extract; LA, lactic acid; PA, propionic acid; WSC, water-soluble carbohydrates.

**FIGURE 4:**
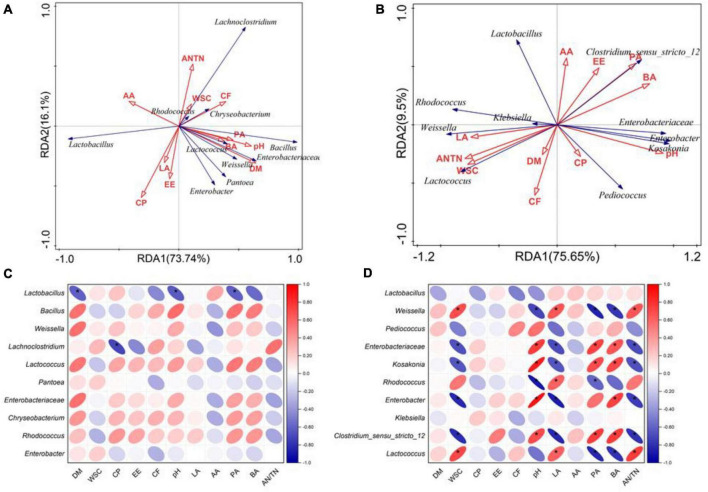
The RDA plots showing the effects of chemical composition and fermentation products on the bacterial community structure at the genus level at flowering **(A)** and maturing stage **(B)**. Spearman correlation heatmap showed the relationship between bacterial classification at the genus level at flowering **(C)** and maturing stage **(D)**, and environmental variables (chemical composition and fermentation products). * indicated the level of significance at *p* < 0.05. AA, acetic acid; AN/TN, ammonia-N in total N; BA, butyric acid; CF, crude fiber; CP, crude protein; DM, dry matter; EE, ether extract; LA, lactic acid; PA, propionic acid; WSC, water-soluble carbohydrates.

As shown in the RDA biplots, a combination of variables explained 99.04 and 98.8% of the variance of the bacterial communities at the phylum level at flowering and maturing stage, respectively (Figures 3A,B). The Partial Mantel test showed that the WSC significantly influenced the bacterial communities at flowering stage. The pH, CP and LA significantly influenced the bacterial communities at maturing stage (Table 5). A combination of variables explained 89.84 and 85.15% of the variance of the bacterial communities at the genus level at flowering and maturing stage, respectively (Figures 4A,B). The Partial Mantel test showed that the DM and EE significantly influenced the bacterial communities at flowering stage, and the LA, CP, PA, pH, and WSC significantly influenced the bacterial communities at maturing stage (Table 5).

**TABLE 5:** The Mantel test (permutations = 999) among chemical composition, fermentation products, and bacterial microbial communities at phylum and genus level at flowering and maturing stage.

	Flowering		Maturing	
		
	*R* ^2^	*P*-value	*R* ^2^	*P*-value
**Phylum level**				
DM	0.021	0.648	0.021	0.314
WSC	0.243	**0.046**	0.013	0.508
CP	0.169	0.172	0.251	**0.002**
EE	0.113	0.112	0.026	0.212
CF	0.029	0.258	0.028	0.300
pH	0.061	0.39	0.105	**0.01**
LA	0.099	0.26	0.452	**0.004**
AA	0.013	0.602	0.041	0.174
PA	0.145	0.228	0.004	0.446
BA	0.074	0.104	0.027	0.306
AN/TN	0.034	0.562	0.031	0.198
**Genus level**				
DM	0.322	**0.028**	0.02	0.352
WSC	0.017	0.67	0.048	**0.046**
CP	0.143	0.108	0.212	**0.008**
EE	0.152	**0.046**	0.024	0.282
CF	0.025	0.446	0.034	0.28
pH	0.056	0.162	0.073	**0.032**
LA	0.071	0.208	0.431	**0.002**
AA	0.083	0.104	0.023	0.324
PA	0.067	0.134	0.107	**0.006**
BA	0.046	0.936	0.01	0.698
AN/TN	0.018	0.556	0.02	0.044

The significance level was tested by the R^2^ and p-values.

DM, dry matter; WSC, water-soluble carbohydrates; CP, crude protein; EE, ether extract; CF, crude fiber; AA, acetic acid; AN, ammonia nitrogen; BA, butyric acid; LA, lactic acid; PA, propionic acid; TN, total nitrogen. Bold values mean p < 0.05.

On phylum level, CF was positively correlated with the relative abundance of *Bacteroidota*, and the relative abundances of *Bacteroidota* and *Actinobacteriota* were positively correlated with WSC, LA, and AN/TN, and negatively correlated with pH, PA, and BA, at flowering stage (Figure 3C). At maturing stage, the relative abundance of *Proteobacteria* was positively correlated with pH and PA, whereas *Firmicutes* was positively correlated with AA (Figure 3D). On genus level, the relative abundance of *Lactobacillus* was negatively correlated with DM, pH and PA, and *Lachnoclostridium* negatively correlated with the CP at flowering stage (Figure 4C). At maturing stage, the relative abundances of *Weissella* and *Lactococcus* were negatively correlated with pH, PA and BA, but positively correlated with WSC, LA, and AN/TN. *Enterobacteriaceae*, *Kosakonia*, *Enterobacter*, and *Clostridium*_*sensu*_*stricto*_12 were negatively correlated with the WSC, LA, and AN/TN, but positively correlated with pH, BA, and PA. The relative abundance of *Rhodococcus* was negatively correlated with pH and PA, but positively correlated with LA (Figure 4D).

## Discussion

### Influence of bloom on ensiled sorghum quality

Sorghum is one of the crops with higher wax coverage, particularly higher wax depositions on its sheath (Peterson et al., 1982). Our results showed that sheath removal of bloom would have a great influence on quality of ensiled sweet sorghum, particularly in WSC and CP, when compared to silage with sheath bloom. For example, bloom reduced the concentrations of WSC for two cultivars at both stages, excepting for an increase for YJ at flowering stage. There was a decrease of CP in sorghum with bloom for two cultivars at two stages, excepting for insignificant changes for JT at flowering stage. On one hand, these results indicated that the bloom would reduce the contents of WSC and CP in ensiled sweet sorghums, thus decreasing the forage quality. This is contrary to the sorghum breeding strategies where high wax coverage has been shown to be positively related to the plant drought tolerance (Peters et al., 2009). On the other hand, the amounts of bloom collected from sheath differed greatly between cultivars and growing stages, which might contribute to the quality differences in the ensiled sorghums.

The fermentation products of ensiled sorghum also differed between sweet sorghums cultivars. For example, the pH level of silages differed in YJ and JT, which might be attributed to the differences of chemical compositions between the sorghum cultivars (Brito et al., 2000). The DM contents of sorghum silage were decreased with ensiling, which is representative of the microbial breakdown of nutrients into water and carbon dioxide. It is observed that a high DM content in YJ, which might slow down the fermentation process, resulting in slow decrease in pH at early stage (Wang et al., 2022). Meanwhile, the bloom decreased the concentrations of lactic acid in YJ at both stages, whereas the content of LA in JT reduced at flowering stage and increased at maturing stage. LA is the principal fermentation product of lactic acid bacterial during ensiled process (Fabiszewska et al., 2019), and their concentrations are generally used to evaluate the quality of the silage (Wang et al., 2019). An increase of LA content would improve the fermentation. However, the cultivars differed in their responses of LA content to bloom. This might be related to the amount of bloom excluded from ensiled sorghum. For example, the amount of bloom of JT was about 1.6 times of YJ at maturing stage. Meanwhile, though the relative abundance of wax compositions was similar among the two cultivars, the actual amounts of wax compositions in leaf, stem and sheath were also differed greatly between two cultivars. This might partly explain the reasons why the bloom increased the LA content in JT at flowering stage, whereas the reverse was true for YJ at both flowering and maturing stage and in JT at maturing stage. Therefore, both quantity and wax compositions on sorghum might influence the fermentation of LAB.

The bloom increased the ratio of ammonia nitrogen in total nitrogen (AN/TN) for YJ at flowering stage, whereas the reverse was true at maturing stage, and a higher AN/TN was observed at maturing stage than at flowering stage. There are varieties of factors influencing the hydrolysis of plant protein during ensiling process (Xie et al., 2021). The bloom might influence the AN/TN through altering the distributions of microbes and thus the enzymes involved in protein hydrolysis (Guo et al., 2015). In the process of fermentation, bacterial growth and reproduction require nitrogen under anaerobic conditions; thus, the content of AN/TN may be the performance of nitrogen reduction. Bloom greatly increased the abundance of *Firmicutes* when compared to removal of bloom treatment. *Firmicutes* is an important acid hydrolytic microbiome, which can generate extracellular proteolytic enzyme (*protease*) for proteolysis (Yin et al., 2021a), thereby affecting AN/TN content. In addition, bloom would inhibit family Enterobacter, which negatively influences fermentation quality by facilitating the formation of ammonia nitrogen and decelerates the acidification of fermentation (Silva et al., 2016). Meanwhile, the nutrition characteristic of sorghum cultivars might also contribute to their difference in fermentation (Fernandes et al., 2020). In this study, the bloom might have reduced the protein degradation for YJ at maturing stage.

Generally, it is difficult to collect all waxes from sorghums using traditional method. Therefore, waxes from leaf, stem and intracuticular wax of sheath might all influence the effects of bloom on sorghum fermentation. However, our results clearly indicated that bloom would influence the fermentation qualities, depending upon the amounts and compositions of bloom, the wax profiles, and the nutrition characteristic of sorghums.

### Influence of sheath bloom on bacterial microbiota

Microorganisms are often influenced by plant cuticular wax, as the presence of cuticular waxes controlling levels of microbial diversity and driving community assembly (Reisberg et al., 2013). Our results showed that bloom significantly decreased Shannon diversity of bacterial when compared with the silage without bloom. This might be possibly brought about by inherent sorghum sheath wax composition to affect microbial communities in their immediate vicinity in process of making silage (Xiao et al., 2020; Ren et al., 2021). Previous researches have indicated that plant wax chemistry components could serve as a selection pressure which would affect the bacteria community composition in the phyllosphere of *Arabidopsis thaliana* and *Zea mays* (Marcell and Beattie, 2002; Reisberg et al., 2013). It was possible that an altered cuticular wax composition might directly influence the microbiota, either by acting as an antimicrobial agent or by providing nutrient for bacteria (Shanker et al., 2007). In this study, bloom wax properties might create niches for specifically adapted bacteria during silage fermentation, which in turn inhibited propagation of other microbes. However, detailed exploration is needed to analyze the interactions between plant wax properties and bacteria communities during silage fermentations.

Silage microbes play an essential role in the biogeochemical processes of ferment, which serves as an important link in ensiled forage quality (Pahlow et al., 2003). Our results showed that the relative abundance of *Firmicutes* phylum was the highest for sorghum silages irrespective of cultivars and harvesting stages. Previous studies have observed that *Firmicutes* is the dominated phylum of bacterial community during silage fermentation in alfalfa (Yang et al., 2019) and sweet corn (Wu et al., 2020b). The bacterial population structure of the sorghum shifted significantly during the ensiling process, and higher abundance of *Proteobacterial* was observed at maturing stage. This suggested that the fermentation process at two growing stages might be different. Bloom relatively increased the abundance of *Firmicutes* but reduced the abundance of *Proteobacteria* at two stages, suggesting that bloom might be related to the significant shift from *Proteobacteria* to *Firmicutes* in the anaerobic acidic ensiling environment, finally resulting in a decrease of structural carbohydrates (Zhao et al., 2016). *Firmicutes* was the dominant species and was beneficial bacteria to ensilage fermentation (Yin et al., 2021a). In the process of anaerobic ensiling, *Firmicutes* is an important acid hydrolytic microbiome, which can generate sufficient extracellular enzymes, including cellulolytic enzyme (cellulase and xylanase) and proteolytic enzyme (protease) (Yin et al., 2021b). The study suggests that bloom might benefit the growth of *Firmicutes* during ensiling, which in turn promoted shifts on bacterial genus from *Proteobacteria* to *Firmicutes*.

At genus level, the combinations of bacterial communities are highly influential on the organic acid profile of silages (Ren et al., 2021). For example, *Weissella* is beneficial bacteria to ensilage fermentation, which is strictly heterofermentative, producing a mixture of LA and AA by metabolizing WSC (Yin et al., 2021a). At maturing stage, the reverse changes of *Weissella* between two cultivars under bloom treatment, which is might attributed to the different wax composition, or microbe on the raw materials. The genus of *Pantoea* and *Enterobacter* are often considered as harmful indicators affecting the ensilage fermentation. *Pantoea* could compete with *Lactobacillus* to ferment sugars to organic acids (e.g., acetic acid and propionic acid) under anaerobic conditions (Raspor and Goranovič, 2008), and the genus of *Enterobacter* competes with LAB for the nutrients, and produces acetic acid as main product. Generally, acetic acid could inhibit the fungal propagation and improve the aerobic stability of silages (Silva et al., 2016). Meanwhile, *Enterobacter* could convert nitrate into nitrite and nitric oxide, and thus suppressing the activity of *Clostridium* (Wu et al., 2020a). Fortunately, at maturing stage, we observed that the relative abundance of *Enterobacter* and *Pantoea* decreased with bloom preserved, whereas the reverse was true for *Weissella* for JT at maturing stage, which was conducive to produce lactic acid and reducing pH (Ni et al., 2018). In this study, bloom may affect the effectiveness of ensiling fermentation, attributing to nutrient competition or interactions in the microbial ecosystem. The bloom might have influenced the growth of microbes in silage. For example, very-long-chain aldehydes promoted *in vitro* pre-penetration processes of *Blumeria graminis* on barley leaves (Hansjakob et al., 2010). Cuticular waxes extracted from mandarin leaves had higher antibacterial activity against *X. citri* (Citrus Canker) (Favaro et al., 2020). Sorghum sheath wax, which was predominated with fatty acids, reduced the growth of *Penicillium* but unchanged *Alternaria alternata* (Xiao et al., 2020). Though the wax compositions might differ in their effects on certain microbe, and even trade-offs might exist between wax compositions. Our results clearly indicated that the bloom wax, which was dominated by series of homologs of fatty acids, significantly influenced the microbes in ensiled sorghums, and thus affecting the silage quality. Furthermore, some *Lactobacillus* strains participated in ensiling would metabolize lactic acid to produce acetic acid under insufficient WSC conditions, resulting in the increase of acetic acid and the decrease of lactic acid (Gallagher et al., 2018; Li et al., 2019).

The sorghum sheath bloom might play an essential role in the biogeochemical processes of fermentation and serve as important links between bacterial communities and silage quality interactions. The RDA analysis of the silage nutritional quality and abundant phyla/genus indicated that CP, WSC, pH, EE, AN/TN, DM, LA, and PA were significantly associated with bacterial community at phyla and genus levels. The far distance among bacterial phylum or genus in RDA indicated that some microbiota can produce various organic acids, transform nitrogen, and change pH, which in turn would influence the silage quality and microbial community. Positive correlations existed between AA, PA, CP, and EE, and these parameters were negatively correlated with LA, AN/TN, and WSC. This relationship may be mutual. For example, WSC can be used easily by bacteria and plays a major role in shaping bacterial community structure (Duniere et al., 2017). During the fermentation process, some microorganisms consume WSC and CP to decompose the cellulose by producing and xylanase and cellulase (Yan et al., 2019). In turn, it is observed that *Lactobacillus* come to dominate by consuming nutrients in silage, and it also dominated at the end of fermentation. This is consistent with many reports on silage bacterial community (Yin et al., 2021a). During the fermentation process, sheath bloom has significant effect on the content of CP, WSC, EE, and CF, this may lead to differences in the fermentation substrate of the bacteria and final bacterial communities. The positive relationship between *Lactococcus* and WSC contents suggested that adequate fermentable substrates could promote the growth of *Lactococcus* during ensiling (Wang et al., 2022). The content of LA was negatively correlated with the abundance of Enterobacter. This is because that *Enterobacter* can ferment lactic acid to acetic acid and other products, reducing the accumulation of lactic acid (Guan et al., 2018), and cause protein degradation (Pahlow et al., 2003). In addition, we observed that pH, content of PA and BA negatively correlated with *Weissella*, whereas content of WSC, LA, and AN/TN positively correlated with *Weissella*. It is possible that *Weissella* could breakdown of nutrients into a mixture of LA and AA, thereby reducing pH and content of PA and BA (Ni et al., 2018). Overall, the study suggests that the difference in silage quality might be attributed to the influence of bloom on microbial community shift (Gallagher et al., 2018).

## Conclusion

As an important secondary metabolite of sorghum plants, the epicuticular wax protects the plants from abiotic and biotic stresses. However, sorghum with bloom harbored distinct fermentation quality and microbial communities. Sheath bloom not only decreased the ensiling quality of sweet sorghum (e.g., increased contents of acetic acid and butyric acid and decreased contents of lactic acid), but also decreased the bacterial Shannon diversity. The abundances of undesirable microbes were suppressed by the bloom, including *Enterobacter*, but that of *Lactobacillus* increased. Decreased contents of lactic acids and bacterial diversity implied that shifts in bacterial community was more important than *Lactobacillus* dominance in hydrolyzing WSC to lactic acids. Such change, then, would influence the fermentation process, the quality of silage and feed-nutritional value. Therefore, our work serves as an important resource for breeding bloom-enriched sweet sorghum cultivars to improve the productivity without at the expense of high-quality silage, thereby satisfying food security and sustainability. In further, detailed evaluation of the influence of bloom on silage qualities and even on livestock digestibility is needed.

## Data availability statement

The data presented in this study are deposited in the NCBI’s Sequence Read Archive repository, accession number PRJNA853257. Available at: https://www.ncbi.nlm.nih.gov/Traces/study/?acc=PRJNA853257&o=acc_s%3Aa.

## Author contributions

YG and WT designed the study. LL, YX, JZ, and HS participated in sample collection. WT performed the experiments and wrote the manuscript with the help of YC, LL, YX, and JZ. YG performed writing - review and edited final manuscript. All authors contributed to the article and approved the submitted version.

## Footnotes

1
https://www.ncbi.nlm.nih.gov

## Abbreviations

AA: acetic acid
AN: ammonia nitrogen
BA: butyric acid
BSTFA: *N,O*-bis(trimethylsilyl)trifluoroacetamide
RDA: redundancy analysis
CP: crude protein
CF: crude fiber
DM: dry matter
EE: ether extract
GC: gas chromatography
LA: lactic acid
LAB: lactic acid bacteria
PA: propionic acid
TN: total nitrogen
WSC: water-soluble carbohydrates.
