# Supplementary material for: Epicuticular wax of sweet sorghum influenced the microbial community and fermentation quality of silage
Source: Front Microbiol. 2022 Jul 29;13:960857. doi: 10.3389/fmicb.2022.960857 (PMC9372506; doi:10.3389/fmicb.2022.960857)
Supplement: Supplementary file 1 [file Data_Sheet_1.docx]

The RNA-Seq reads were deposited and available at the database Sequence Read Archive at NCBI (https://www.ncbi.nlm.nih.gov) under the master accession number of Bioproject PRJNA853257, and the accession number for each RNA-Seq data is provided in the table 1.

Table 1 Metadata data accession, sample name and treatment at NCBI

| Data files | NCBI Accession # | Samples | Treatment |
| --- | --- | --- | --- |
| raw.split.JT0_1.1.fq  raw.split.JT0_1.2.fq | SRR19860148 | JT0_1 | Jintian without bloom at flowering stage |
| raw.split.JT0_2.1.fq  raw.split.JT0_2.2.fq | SRR19860147 | JT0_2 |  |
| raw.split.JT0_3.1.fq  raw.split.JT0_3.2.fq | SRR19860136 | JT0_3 |  |
| raw.split.JT0_A.1.fq  raw.split.JT0_A.2.fq | SRR19860131 | JT0_A | Jintian without bloom at maturing stage |
| raw.split.JT0_B.1.fq  raw.split.JT0_B.2.fq | SRR19860130 | JT0_B |  |
| raw.split.JT0_C.1.fq  raw.split.JT0_C.2.fq | SRR19860129 | JT0_C |  |
| raw.split.JT1_1.1.fq  raw.split.JT1_1.2.fq | SRR19860128 | JT1_1 | Jintian with bloom at flowering stage |
| raw.split.JT1_2.1.fq  raw.split.JT1_2.2.fq | SRR19860127 | JT1_2 |  |
| raw.split.JT1_3.1.fq  raw.split.JT1_3.2.fq | SRR19860126 | JT1_3 |  |
| raw.split.JT1_A.1.fq  raw.split.JT1_A.2.fq | SRR19860125 | JT1_A | Jintian with bloom at maturing stage |
| raw.split.JT1_B.1.fq  raw.split.JT1_B.2.fq | SRR19860146 | JT1_B |  |
| raw.split.JT1_C.1.fq  raw.split.JT1_C.2.fq | SRR19860145 | JT1_C |  |
| raw.split.YJ0_1.1.fq  raw.split.YJ0_1.2.fq | SRR19860144 | YJ0_1 | Yajin without bloom at flowering stage |
| raw.split.YJ0_2.1.fq  raw.split.YJ0_2.2.fq | SRR19860143 | YJ0_2 |  |
| raw.split.YJ0_3.1.fq  raw.split.YJ0_3.2.fq | SRR19860142 | YJ0_3 |  |
| raw.split.YJ0_A.1.fq  raw.split.YJ0_A.2.fq | SRR19860141 | YJ0_A | Yajin without bloom at maturing stage |
| raw.split.YJ0_B.1.fq  raw.split.YJ0_B.2.fq | SRR19860140 | YJ0_B |  |
| raw.split.YJ0_C.1.fq  raw.split.YJ0_C.2.fq | SRR19860139 | YJ0_C |  |
| raw.split.YJ1_1.1.fq  raw.split.YJ1_1.2.fq | SRR19860138 | YJ1_1 | Yajin with bloom at flowering stage |
| raw.split.YJ1_2.1.fq  raw.split.YJ1_2.2.fq | SRR19860137 | YJ1_2 |  |
| raw.split.YJ1_3.1.fq  raw.split.YJ1_3.2.fq | SRR19860135 | YJ1_3 |  |
| raw.split.YJ1_A.1.fq  raw.split.YJ1_A.2.fq | SRR19860134 | YJ1_A | Yajin with bloom at maturing stage |
| raw.split.YJ1_B.1.fq  raw.split.YJ1_B.2.fq | SRR19860133 | YJ1_B |  |
| raw.split.YJ1_C.1.fq  raw.split.YJ1_C.2.fq | SRR19860132 | YJ1_C |  |
