# Supplementary material for: Epicuticular wax of sweet sorghum influenced the microbial community and fermentation quality of silage
Source: Front Microbiol. 2022 Jul 29;13:960857. doi: 10.3389/fmicb.2022.960857 (PMC9372506; doi:10.3389/fmicb.2022.960857)
Supplement: Supplementary file 2 [file Table_1.docx]

**Supplementary material**

**Epicuticular wax of sweet sorghum influenced the microbial community and fermentation quality of silage**

**TABLE S1** Days from sowing to flowering (BBCH 63) and maturing (BBCH 85) of the two tested sorghum cultivars.

| Cultivars | Development period | |
| --- | --- | --- |
|  | sowing to flowering | sowing to maturity |
| Yajin 2 | 56 | 100 |
| Jintian | 56 | 98 |
